# Hope and challenge: Precision medicine in bladder cancer

**DOI:** 10.1002/cam4.1979

**Published:** 2019-03-24

**Authors:** Hongwei Su, Haitao Jiang, Tao Tao, Xing Kang, Xu Zhang, Danyue Kang, Shucheng Li, Chengxi Li, Haifeng Wang, Zhao Yang, Jinku Zhang, Chong Li

**Affiliations:** ^1^ Department of Urology Affiliated Traditional Chinese Medicine Hospital Southwest Medical University Luzhou China; ^2^ Core Facility for Protein Research Institute of Biophysics Chinese Academy of Sciences Beijing China; ^3^ School of Medical Laboratory Science and Biotechnology Taipei Medical University Taipei China; ^4^ Department of Urology The Affiliated Luohu Hospital of Shenzhen University Shenzhen University Shenzhen China; ^5^ Michigan State University East Lansing Michigan; ^6^ Department of Urology The Second Affliated Hospital of Kunming Medical University Kunming China; ^7^ College of Life Science and Technology Beijing University of Chemical Technology Beijing China; ^8^ Department of pathology First Central Hospital of Baoding Baoding Hebei China; ^9^ Beijing Jianlan Institute of Medicine Beijing China

**Keywords:** bladder cancer, gene therapy, immunotherapy, precision medicine

## Abstract

Bladder cancer (BC) is a complex disease and could be classified into nonmuscle‐invasive BC (NMIBC) or muscle‐invasive BC (MIBC) subtypes according to the distinct genetic background and clinical prognosis. Until now, the golden standard and confirmed diagnosis of BC is cystoscopy and the major problems of BC are the high rate of recurrence and high costs in the clinic. Recent molecular and genetic studies have provided perspectives on the novel biomarkers and potential therapeutic targets of BC. In this article, we provided an overview of the traditional diagnostic approaches of BC, and introduced some new imaging, endoscopic, and immunological diagnostic technology in the accurate diagnosis of BC. Meanwhile, the minimally invasive precision treatment technique, immunotherapy, chemotherapy, gene therapy, and targeted therapy of BC were also included. Here, we will overview the diagnosis and therapy methods of BC used in clinical practice, focusing on their specificity, efficiency, and safety. On the basis of the discussion of the benefits of precision medicine in BC, we will also discuss the challenges and limitations facing the non‐invasive methods of diagnosis and precision therapy of BC. The molecularly targeted and immunotherapeutic approaches, and gene therapy methods to BC treatment improved the prognosis and overall survival of BC patients.

## 1 INTRODUCTION

Bladder cancer (BC) is a common urinary malignant tumor.^1^ In 2012, bladder cancer ranks as the ninth most common‐diagnosed cancer with estimated 430,000 new cases.^1^, ^2^ BC is the most prevalent urinary cancer in China, the ratio of the incidence BC for males to females and cities to rural areas is 3.3 and 2.4, respectively. For the past few years, the overall incidence and mortality of BC have shown a gradual upward trend and become a serious threat to the health of people.^1^, ^2^, ^3^

With the development of minimally invasive urology technique and a new initiative on precision medicine, the treatment of BC enters the epoch of precision medicine. Precision medicine, also called personalized medicine, is a novel medical concept. The cross‐application of genome sequencing, proteomics technology, bioinformatics, and big data analysis are applied to investigate the etiological factors, precise classification, and therapeutic targets of disease at the molecular level, ultimately actualize precise personalized medicine, and improve the prophylactic and therapeutic effect of diseases.^4^, ^5^ In 2015, former U.S. president Obama lodged “precision medicine initiative” and intended to make precision medicine concept penetrating into various medical fields. They have invested a mass of resources in the project to carry out in‐depth investigations. Therefore, precision medicine is the general trend of medical development.^6^

## 2 RESEARCH FRONT OF PRECISION DIAGNOSIS IN BLADDER CANCER

### 2.1 Traditional diagnostic approach

Major diagnosis methods of BC are imaging tests (ultrasound, CT, MRI), cystoscopy, and urine cytology presently (Table 1a). These examinations have high accuracy in patients with advanced BC and biopsy is the gold standard for bladder cancer screening. Urine cytology is a non‐invasive method. However, it has poor sensitivity and might be influenced by factors such as kidney stone, renal inflammation, and hemorrhage. Cystoscopy is an invasive technique that may miss diagnose small tumors, carcinoma in situ (CIS), and inflammatory carcinoma. Therefore, finding the precise, non‐invasive, painless, economical, and convenient methods of early diagnosis for BC has become a hotspot in recent years.

**Table 1:** A summary of modality in the diagnosis of bladder cancer

Method	Description	Pros	Cons	Refs
(a) Traditional diagnostic approaches				
Urine cytology	Using a microscope to look for cancer cells in urine	This examination is of high accuracy for patients with advanced bladder cancer	Not finding cancer on this test does not always mean patients are cancer free	ACS
Ultrasound	It uses sound waves to create images of internal organs	It can be useful in determining the size of a bladder cancer and whether it has metastasis	The accuracy is poor when flat, plaque like tumors are present, negative test result does not mean the absence of tumor	ACS
CT	It uses x‐rays to make detailed cross‐sectional images of patients’ body	It can deliver thorough information about the size, shape, and location of tumors in the urinary tract and bladder. It is also helpful in showing swollen lymph nodes that might contain cancer	The test is unreliable to detect the flat lesions and carcinoma in situ. It has limited capability in distinguishing bladder muscle layers	ACS^81^
MRI	It can show detailed images of soft tissues in the body	It has excellent soft tissue resolution and multiplanar capabilities and particularly helpful to show whether the cancer has metastasize into adjacent tissues or lymph nodes	MRI can cost a lot	ACS^81^
Cystoscopy	Doctors use cystoscopy —a fine tube with a tiny light and lens on the end and insert it into the bladder through urethra	It is the best way to find bladder cancer at present	It is an invasive inspection and it is easy to cause misdiagnose in small tumor, carcinoma in situ, inflammatory carcinoma which could not be seen by naked eye	ACS
(b) Tumor markers in bladder cancer				
BTA tests	These tests detect the urine bladder tumor‐associated antigen (BTA), also called CFHrp	The combination of a variety of tumor markers has high‐sensitivity. Non‐invasive	No single marker coincidentally has high sensitivity and specificity for the diagnosis of bladder cancer	ACS
Immunocytochemistry	A common laboratory technique looks for cells in the urine that have substances like mucin and carcinoembryonic antigen (CEA) that usually exist on cancer cells	The combination of a variety of tumor markers has high‐sensitivity. Non‐invasive	No single marker coincidentally has high sensitivity and specificity for the diagnosis of bladder cancer	ACS, 7‐15
NMP22 test	The test detects a bladder cancer marker called nuclear matrix protein 22(NMP22) in the urine	The combination of a variety of tumor markers has high‐sensitivity. Non‐invasive	No single marker coincidentally has high sensitivity and specificity for the diagnosis of bladder cancer	ACS
(c) New imaging technology				
PET/CT	A new imaging equipment that combines the functional metabolic imaging of PET and the structural imaging of CT	It can make whole‐body imaging applied for patients with suspected bladder cancer recurrence, which showed a high accuracy in early diagnosis	The diagnostic ability of PET/CT for primary lesion and detrusor involvement is difficult to assess in a small number of patients	17, 18, 19, 20, 21, 90
ICG‐pHLIP target imaging	It uses a kind of membrane‐bound peptide which can specifically target acidic cells both in vitro and in vivo	It can specifically recognize advanced urothelial carcinoma (include muscle‐invasive and nonmuscle‐invasive)	TURBT (Transurethral resection of bladder tumor) will induce false‐positive in ICG‐pHLIP target imaging	22, 23
(d) New endoscopic imaging technique				
Fluorescence cystoscopy (also known as blue light cystoscopy)	A photo‐activated agent that can be taken up by cancer cells is put into the bladder during cystoscopy. By illuminating the bladder with blue light through cystoscopy, cells containing the drug will glow (fluorescence)	This can assistant the doctor finding abnormal tissue that might have been missed under white light cystoscopy	Expensive equipment	ACS^24^, ^25^, ^26^, ^27^, ^28^
(e) Immunological diagnostic technique	It uses the number of immune cells and the concentration of related cytokines in tumor microenvironment to predict the prognosis of bladder cancer	Non‐invasive	Most co‐stimulate molecules of inflammatory cells and tumor cells as well as cytokines in serum can be used as the predictors of patient prognosis only in the single factor analysis	37, 38, 39

ACS, American Cancer Society.

### 2.2 Tumor markers in diagnosis of bladder cancer

The urinary tumor markers and methods approved by FDA were used for BC diagnosis including bladder cancer antigen (BTA), nuclear matrix protein 22 (NMP22), fibrin/fibrinogen degradation products (FDP), Immunocytochemistry, Fluorescent *in situ* hybridization (FISH), etc.^7^ Bell et al suggested that the high expression of NMP22 in urine was related to a decreased recurrence‐free survival and progression‐free survival (PFS) in bladder cancer concurrently.^8^ A study of Yafi et al indicated that the combination of urine cytology and NMP22 could effectively improve the rate of early diagnosis of high‐grade tumor.^9^ Li et al addressed that AG‐α3β1 (the antigen recognized by BCMab1) was closely related to the progression and prognosis of BC, and could be used as a biomarker for early diagnosis or postoperative recurrence of BC.^10^, ^11^

Furthermore, the biomarkers in early diagnosis and postoperative monitoring of BC include telomerase, hyaluronic acid (HA), hyaluronidase (HAase), microRNA, long noncoding RNA (lncRNA), DNA methylation, survivin, microsatellite alteration (MA), cell‐free plasma DNA (cfp‐DNA), and circulating tumor cell (CTC).^12^, ^13^, ^14^ Roperch et al pointed out DNA methylation combined with the mutation status of *FGFR3* resulted in an accurate diagnosis for NMIBC with a specificity and sensitivity of 97.6% and 84.8%, respectively.^15^ Martens‐Uzunova et al emphasized that lncRNA could be used as a novel non‐invasive tumor marker for the diagnosis of urinary tumor.^16^

### 2.3 New imaging technology

Imaging techniques are of great consequence in the accurate diagnosis of BC, which include multi‐slice spiral CT, transurethral ultrasound of bladder, positron emission tomography/computed tomography (PET/CT), and target imaging.^17^, ^18^, ^19^, ^20^, ^21^, ^22^, ^23^ FDG PET/CT exhibited a significant prognostic value in assessing progression‐free survival and overall survival, which was validated in patients with suspected recurrent BC.^20^ Meta‐analysis showed that PET/CT could accurately monitor the metastasis of BC. However, it is difficult to assess the primary lesion of BC patients by PET/CT.^21^

pH low insertion peptides (pHLIP, Table 1c), a membrane‐bound peptide, can specifically target acidic cells both in vitro and in vivo when cells are in a low pH environment.^22^ The study of Golijanin et al demonstrated that ICG‐pHLIP targeted imaging could specifically recognize advanced urothelial carcinoma (including MIBC and NMIBC) and improve the early diagnosis of BC, which provided a novel alternative for the diagnosis and treatment of BC.

### 2.4 New endoscopic imaging technique

New endoscopic imaging techniques for the diagnosis of BC mainly include fluorescence cystoscopy, narrow spectrum optical cystoscopy, optical coherence tomography and confocal laser endoscopy, etc. (Table 1d). With the help of photosensitizer, such as 5‐ALA and its derivative HAL, the fluorescence cystoscopy has a higher diagnostic rate for CIS, flat or papillary cancerous nidus. Its intraoperative application can improve the overall resection rates of the tumor.^24^, ^25^, ^26^ A multicenter research reported by Palou et al pointed out that with the assist of blue light, white light cystoscopy would have an obviously elevated rate of early diagnosis of NMIBC (especially for CIS and Ta stage).^27^ Pan et al found that more than 80% BC expressed CD47, and fluorescence cystoscopy with CD47 targeted molecular imaging could improve the diagnostic rate of BC and overall tumor resection.^28^ Development and clinical application of new endoscopic techniques provide more diagnostic strategies and options for BC. However, due to the expensive equipment, it has not yet been widely used.

### 2.5 Gene and genomics

Recently, the molecular classification of tumor cells is expected to cultivate the accuracy of BC diagnosis. Choi et al divided MIBC into three molecular subtypes; basal type, luminal type, and p53‐like type. Basal type was characterized by p63 activation, squamous differentiation, and invasive activity. Luminal type was characterized by PPARγ activation, estrogen receptor transcription activated by *FGFR3* mutation. p53‐like type was resistant to neoadjuvant chemotherapy agents (methotrexate, vincaleukoblastine, azithromycin, cisplatin, etc.), and all the chemoresistant bladder cancer showed p53‐like subtype after chemotherapy.^29^ In a TCGA analysis, urothelial carcinoma was divided into four genotypes: type I and type II cells have high expression of ERBB2 and activation of ESR2 pathway, whereas type I has *FGFR3* mutation and a papillary morphology. Type III cells have squamous cells and stem cells properties (increased expression of EGFR and keratin). Type IV is between Type II and III.^30^ A study suggested that the expression signature of *ANXA10, DAB2, HYAL2, SCD1,* and *MAP4K1* could predict progression of T1G3 BC with the sensitivity of 79% and the specificity of 86%.^31^

Telomerase reverse transcriptase (*TERT*) promoter mutation can up‐regulate *TERT* expression and enhance the tumor invasibility. A study of somatic *TERT* promoter mutations in 302 patients with genitourinary tumors suggested that urothelial carcinoma had the highest *TERT* promoter mutation rates and no *TERT* promoter mutations were found in prostate cancer, which demonstrated *TERT* promoter mutation could be used as a target for the monitoring of recurrence and treatment of BC.^32^ The study of Li et al demonstrated that there was a high frequency of *TERT* promoter C228T mutation in bladder cancer stem cells (BCSCs). The expression of *TERT* positively correlated with the progression and prognosis of BC.^33^

The methylation of histone H3K4me3, and the expression of downstream *GATA4*,* ETS1* were enhanced significantly in recurrent BC tissues and patients with higher expression of *GATA4* and *ETS1* has a shorter survival time, which revealed that H3K4me3 and the expression of GATA4 and ETS1 were promising targets for monitoring and treatment of BC recurrence.^34^ Yang et al identified 21 key altered genes in BCSCs including five novel mutated genes (*GPRC5A, MKL1, PAWR, PITX2,* and *RGS9BP*) in BC by single‐cell sequencing, and found that the co‐mutations of *ARIDA1, GPRC5A,* and *MLL2* played a critical role in maintaining the stemness of bladder cancer stem cells.^35^, ^36^ Above results provided more possibilities for the targeted therapy of BC.

### 2.6 Immunological diagnostic technique

Cells in a microenvironment (Table 1e) can be induced by tumor cells to produce a large number of inflammatory factors, growth factors, chemokines as well as proteolytic enzymes, which promotes the proliferation, invasion, and metastasis of tumor cells.^37^, ^38^ Recent studies demonstrated that the number of immune cells and the concentration of related cytokines in the tumor microenvironment could be used as prognostic predictors of BC.^39^, ^40^, ^41^ For instance, Mbeutcha et al revealed that the neutrophils to lymphocyte ratio was associated with cancer progression and occurrence, so the application of neutrophils to lymphocyte ratio as a biomarker was helpful to improve the accuracy in predicting postoperative recurrence and progression of tumors.^41^ A meta‐analysis showed that high C‐reactive protein could be used as an independent predictor of mortality in invasive bladder cancer patients.^40^ Margel et al suggested that HSP60 and IL‐13 secreted by tumor cells in urine or tumor microenvironment could be used as biomarkers for diagnosis and staging of BC.^39^

The immune cells and cytokines in the tumor microenvironment showed great diagnostic and prognostic value for recurrence and progression of tumor. The detection of immune cells and cytokines in urine or blood will provide new modalities for BC diagnosis, and more accurate typing of BC will be the focus of future research.

## 3 PROGRESSES IN PRECISION THERAPY OF BLADDER CANCER

BC can be divided into nonmuscle‐invasive bladder cancer (NMIBC): Tis, Ta, T_1_ (Table 2), and muscle‐invasive bladder cancer (MIBC): T2 stage and higher. Currently, the first choice for NMIBC treatment in clinic is transurethral resection of bladder tumor plus postoperative adjuvant intravesical instillation (chemotherapy and immunotherapy). The first choice for the MIBC treatment is radical cystectomy plus pelvic lymphadenectomy. With the development of minimally invasive treatment and deep knowledge on molecular mechanisms of carcinogenesis and progression of BC, a large number of new therapies have emerged in the clinic, which improved the accuracy of BC treatment and prognosis.

**Table 2:** Current bladder cancer TNM staging^a^ (Revised by ACS in 2016)

T categories for bladder cancer
TX: Main tumor cannot be assessed due to lack of information
T0: No evidence of a primary tumor
Ta: Non‐invasive papillary carcinoma
Tis: Non‐invasive flat carcinoma (flat carcinoma in situ, or CIS)
T1: The tumor has grown from the layer of cells lining the bladder into the connective tissue below. It has not grown into the muscle layer of the bladder
T2: The tumor has grown into the muscle layer
T2a: The tumor has grown only into the inner half of the muscle layer
T2b: The tumor has grown into the outer half of the muscle layer
T3: The tumor has grown through the muscle layer of the bladder and into the fatty tissue layer that surrounds it
T3a: The spread to fatty tissue can only be seen by using a microscope
T3b: The spread to fatty tissue is large enough to be seen on imaging tests or to be seen or felt by the surgeon
T4: The tumor has spread beyond the fatty tissue and into nearby organs or structures. It may be growing into any of the following: the stroma (main tissue) of the prostate, the seminal vesicles, uterus, vagina, pelvic wall, or abdominal wall
T4a: The tumor has spread to the stroma of the prostate (in men), or to the uterus and/or vagina (in women)
T4b: The tumor has spread to the pelvic wall or the abdominal wall
N categories for bladder cancer (regional lymph nodes)
NX: Regional lymph nodes cannot be assessed due to lack of information
N0: There is no regional lymph node spread
N1: The cancer has spread to a single lymph node in the true pelvis
N2: The cancer has spread to two or more lymph nodes in the true pelvis
N3: The cancer has spread to lymph nodes along the common iliac artery
M categories for bladder cancer
M0: There are no signs of distant spread
M1: The cancer has spread to distant parts of the body

a Stages: Stage I (T1, N0, M0); Stage II (T2a or T2b, N0, M0); Stage III (T3a, T3b, or T4a, N0, M0); Stage IV (Any T, N1 to N3, M0 or Any T, any N, M1).

### 3.1 Minimally invasive precision treatment technique

Emerging transurethral plasmakinetic resection, transurethral laser surgery (holmium laser, thulium laser, green laser, neodymium laser) were associated with better safety and effectiveness than traditional TURBT in the treatment of NMIBC.^42^, ^43^, ^44^, ^45^ Scholars suggested that transurethral bipolar plasmakinetic resection and transurethral holmium laser surgery were more effective than conventional monopolar TURBT for the treatment of NMIBC.^46^ However, the large‐sample randomized controlled trials (RCT) of urethral laser surgery for NMIBC is rare, and the evidence was not sufficient to support its safety and efficacy.

Fluorescence cystoscopy (FC) and narrow band imaging (NBI) are increasingly concerned by researchers. Witjes et al suggested that if possible, all the patients should receive fluorescence cystoscopy at the first TURBT treatment, which was especially applicable for patients with CIS and positive urinary cytology results.^47^ In a RCT involving 185 NMIBC patients, patients with fluorescence cystoscopy required less short‐term mitomycin chemotherapy and has a higher diagnostic rate for recurrent CIS (26%) than white light cystoscopy group (77%). However, the tumor recurrence rates had no significant difference between the two groups.^48^ Naito et al pointed out that NBI‐assisted TURBT could effectively reduce the recurrence rates of low‐risk NMIBC patients.^49^ Fluorescence and NBI cystoscopy can solve the problem of easy omission of small lesions under white light cystoscopy and improve the accuracy of NMIBC minimally invasive treatment.

Radical cystectomy is the standard treatment of MIBC, which can effectively avoid local recurrence and distant metastasis. Radical cystectomy includes conventional open surgery, 2‐D laparoscope, 3‐D laparoscope, and robot‐assisted radical cystectomy (RARC).^50^, ^51^, ^52^ Simone et al successfully implemented the RARC plus intracorporeal neobladders plus extended pelvic lymph node dissection in 45 BC patients and showed the safety and effectiveness of RARC in MIBC treatment.^53^ The high flexibility and accuracy of RARC indicated that robotic surgery would be the future direction for the development of urology surgery.

### 3.2 Immunotherapy and biomarkers for curative effect prediction

Perfusing immunologic agents through bladder can induce immune response, and prevent tumor recurrence and progression. Bacillus Calmette‐Guérin (BCG), an attenuated vaccine primarily applied on prevention of tuberculosis, is the first choice for bladder instillation after operation of high‐risk NMIBC.^54^, ^55^ Nevertheless, the results of a RCT indicated that maintenance of BCG instillation for 3 years did not result in a significant reduction in recurrence and progression rates of high‐risk NMIBC patients.^56^ The insensitivity of some patients to BCG perfusion and high adverse drug reactions limits its popularization and application in clinical treatment. The selection of sensitive patients for BCG therapy as well as the timing, course, and dosage of bladder perfusion is the focus of this study.

Kamat et al found that BC patients with high concentration of urinary IL‐2, IL‐8, and IL‐18 had a low recurrence probability after BCG treatment. A prediction model of recurrence (CyPRIT) could timely and effectively modify the dosage and duration of BCG perfusion.^57^ Ryk et al found that the prognosis of patients with *NOS2* promoter microsatellite could not be improved by BCG immunotherapy. However, patients with *NOS3*‐rs2070744 and *NOS3*‐rs1799983 genotypes showed lower tumor specific mortality and progression rates after BCG treatment. Therefore, gene polymorphisms could be used as potential biomarkers for predicting the efficacy of BCG immunotherapy.^58^

Dendritic cells dysfunction is one of the major causations of BC, and the increase in the number and activity of mature DCs in tumor tissue by DC vaccine and other approaches can enhance the immune killing effect on tumor.^59^ Wang et al indicated that B7‐H1 silencing could enhance the antitumor effect of bladder cancer by antigen‐expression DC, which provided a new strategy for BC immunotherapy.^60^ Aside from BCG and DC vaccines, there are numerous reports on the use of immunotoxin coupled mAb, IFN‐α, IL‐2, etc. in BC immunotherapy.^61^, ^62^

### 3.3 Chemotherapy

#### 3.3.1 Postoperative adjuvant chemotherapy

Bladder instillation chemotherapy is of great significance for reducing the postoperative recurrence rates of NMIBC. At present, the common drugs used for bladder instillation chemotherapy are pirarubicin, epirubicin, doxorubicin, mitomycin C, etc.^63^ Perfusion methods include immediate postoperative perfusion and maintenance perfusion. Immediate postoperative perfusion can prevent tumor cell implantation. Maintenance perfusion can reduce the recurrence rate of tumor, but no evidence showed it could impede the progression of tumor. A meta‐analysis revealed that immediate perfusion of single chemotherapeutic agent could reduce the risk of recurrence in pTa~pT1 stage BC.^64^ EAU's latest guideline recommends that low‐risk and moderate‐risk NMIBC patients should receive postoperative adjuvant intravesical chemotherapy, low‐risk and low recurrence rates moderate‐risk NMIBC patients were recommended to perfuse immediately after operation. Patients with moderate NMIBC should receive a 1‐year maximum dose of chemotherapy (or BCG) maintenance perfusion after operation.^65^

#### 3.3.2 Neoadjuvant chemotherapy

Neoadjuvant chemotherapy aims to control the local lesion and distant small tumor metastasis, reduce the difficulty of surgery, and improve the postoperative long‐term survival rate of patients. However, the optimal operation time will be delayed in patients who are insensitive to neoadjuvant chemotherapy.^66^ The neoadjuvant cisplatin‐based combination chemotherapy is the standard strategy for invasive bladder cancer. Currently, the manner routinely used is GC (gemcitabine plus cisplatin) scheme and MVAC (methotrexate, vincristine, azithromycin, and cisplatin) scheme. In a study reported by Galsky, the pathologic complete response (pCR) between the two schemes have no statistical significance (*P *= 0.77) which demonstrated that the clinical efficacy of GC and MVAC cohort is equivalent.^67^ A meta‐analysis indicated the GC cohort and MVAC cohort had similar pCR in retrospective study. However, a prospective randomized study showed that the MVAC cohort had a higher pCR rate. This discrepancy should be confirmed by more large‐sample prospective randomized studies.^68^

The molecular markers might be used for predicting the sensitivity of neoadjuvant chemotherapy of BC including *ERBB2*,* Ki‐67*,* BRAC1*,* MDR1*,* ERCC1*,* GDPD3*,* SPRED1*, etc.^69^, ^70^, ^71^, ^72^, ^73^ With the development of neoadjuvant chemotherapy agents, preservation treatment could be considered for BC patients who are sensitive to neoadjuvant chemotherapy. The identification of molecular predictive biomarkers of tumor responsiveness to neoadjuvant chemotherapy is essential to practice precise medicine in patients with MIBCs.

### 3.4 Gene therapy

The majority of human epithelial cells, such as BC cells, express the coxsackie/adenovirus receptor (CAR) and are infectable by adenoviruses. The restoration of the normal function of tumor‐suppressor genes in BC is a reasonable approach of targeted gene therapy for bladder cancer with replication‐defective adenoviral vectors in vivo.^74^, ^75^

For example, *p53* frequently altered in BC, and *p53* gene transfer mediated by adenovirus is growth‐inhibitory to BC cells in vitro. The vector Ad5CMV‐P53 containing human wild‐type *p53* has been administered intravesically to BC patients in a phase I clinical trial. *P53* gene transfer inhibited the growth, increased the sensitivity to chemotherapy drugs of BC cells and showed no apparent toxicity to normal tissues.^76^

Retinoblastoma (*RB*) gene also frequently mutated in BC, and the restoration of wild‐type RB expression is growth‐inhibitory to BC cells.^77^ CG‐0700, a recombinant adenovirus specifically targeting Rb pathway, could replicate selectively in tumor cells and produce granulocyte macrophage‐colony stimulating factor (GM‐CSF).^78^, ^79^ GM‐CSF could recruit and mature myeloid cells and enhance the local antitumor activity. In the clinic, CG‐0700 led to a complete response rate of 48.6% at 10.4 months without adverse side effects.^78^

rAd‐IFN/Syn‐3 (Instiladrin), a nonreplicating recombinant adenovirus vector, contained the human IFN alpha‐2b (*IFN*α*2b*) gene. The results of the phase I and II trials indicating that rAd‐IFN/Syn‐3 could lead to detectable levels of IFN‐α in urine and the recurrence‐free survival was 35% at 12 months in the phase II trial (Table 3).^80^, ^81^

**Table 3:** Summary of gene therapy for bladder cancer

Agent	Study type	Study design	Patient disease status	Primary outcome	Trial ID	Study status
CG‐0700	Phase III	Nonrandomized—single arm	High grade Ta, T1, or Tis	DCR at 18 mo	NCT02365818	Active, not recruiting
rAD‐IFN/Syn3	Phase III	Nonrandomized—single arm	High grade Ta, T1, or Tis	EFS at 12 mo	NCT02773849	Actively recruiting
VPM1002BC	Phase I/II	Phase 1: Induction: 6 intravesical instillations in 6‐12 wk (dose de‐escalation) Phase 2: Induction: as phase 1 maintenance: 3 instillations at months 3, 6, and 12	High grade Ta, T1, or Tis	Safety, tolerability, RFS at 60 wk	NCT02371447	Actively recruiting

DCR, durable complete response; EFS, event‐free survival; RFS, recurrence‐free survival.

More and more novel gene therapies are currently being tested in clinical trials for BC. Adenovirus‐mediated gene transfer in vivo may be most appropriate for a microscopic tumor burden such as that involved in minimal residual disease or CIS. Furthermore, it will be pivotal to utilize molecular subtyping of BC in designing future studies and analyzing the response of different subtyping patients to distinct therapeutic regimens.^82^ The ongoing efforts in this field would be very encouraging, and the outlook for finding more effective and specific treatments look very promising.

### 3.5 Targeted therapy

Targeted therapy utilizes the agent to recognize oncogenic sites (cell surface antigen, membrane protein molecule, or gene fragment) of tumor cells, and then induces tumor cells necrosis and apoptosis. The mAb (MPDL3280A) developed by Powles et al inhibits the binding of PD‐L1 with PD‐1 and CD80, and has less adverse drug reaction and nephrotoxicity compared with the traditional chemotherapy agents. MPDL3280A has been approved by the US FDA for the treatment of BC.^83^

AG‐α3β1 (BCMab1 antigen) expressed specifically on the surface of bladder cancer cells. BCMab1 is an anti‐AG‐α3β1 mAb, which can inhibit growth, proliferation, invasion, metastasis of tumors, and enhance the immune response to BC. Furthermore, AG‐α3β1 could be used as a target for targeted therapy of BC. Moreover, BCMab1 can inhibit the growth of orthotopic transplantation tumor in nude mice without obvious toxic and side effect, which preliminarily proved the safety and feasibility of BCMab1 for targeted therapy of BC.^10^, ^84^

**Figure 1:**
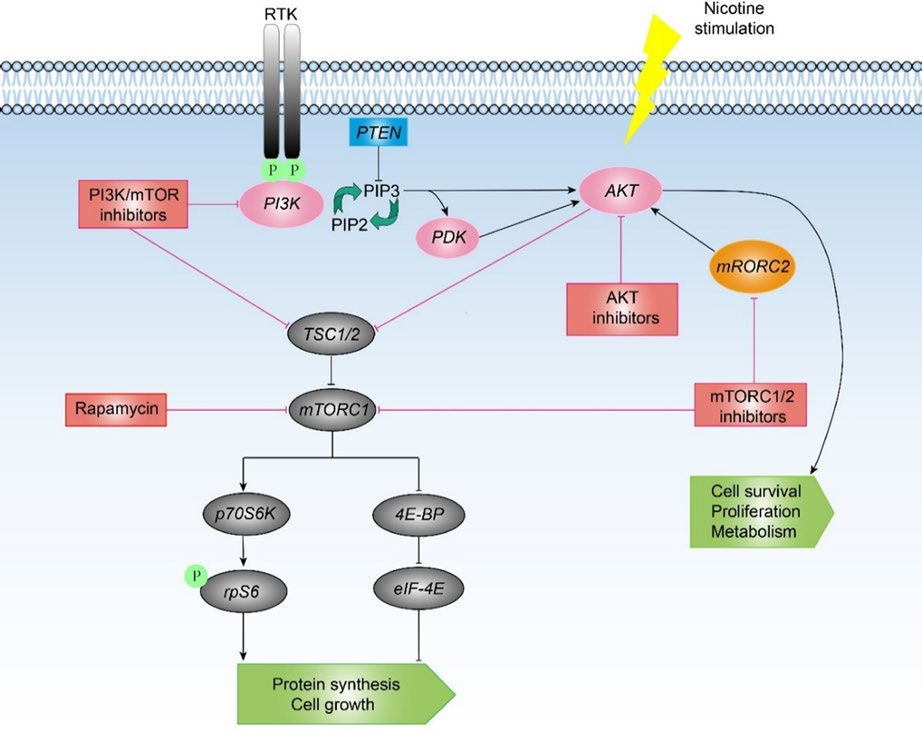
PI3K/Akt /mTOR pathway

PI3K/Akt/mTOR pathway (Figure 1) is closely related to the tumorigenesis and progression of BC and plays a critical role in the apoptosis, survival and cell‐cycle of tumor cells. Yuge et al suggested nicotine could induce the growth and chemotherapy resistance of bladder cancer cells by activating the PI3K/Akt/mTOR pathway.^85^ Costa et al found that PI3K/Akt/mTOR pathway related signaling molecules (pAkt, pmTOR, pS6) overexpression could obviously reduce the tumor‐specific survival rate of patients. Rapamycin (mTOR pathway inhibitor) can diminish the invasion of tumor cells by down‐regulating the expression of pS6, which demonstrated that the inhibitors of mTOR pathway have a broad application in the remedy of BC.^86^

Currently reported potential therapeutic targets of BC included transcription factors (TP53, EP300, E2F3/SOX4, MDM2, CREBBP, NCOR1), histone modification molecules (ARID1A, MLL2), cell‐cycle regulatory molecules (RB, CDKN1A, CDKN2A), gene integration related molecules (ERCC2, STAG), RTK signaling pathway (FGFR3), EGFR pathway (EGFR, EGRBB2, EGRBB3), hedgehog pathway (GLI1, GLI2, JAG2), KMT1A‐GATA3‐STAT3 pathway, etc.^30^, ^87^, ^88^, ^89^ To cultivate accurate bladder cancer targeted therapy, further studies should focus on develop targeted agents with high specificity, high sensitivity, and little adverse drug reaction. In Table 4 we have listed the potential therapeutic targets for bladder cancer and the signaling pathways for these targets.

**Table 4:** Potential therapeutic targets and signaling pathway for bladder cancer

Target	Signaling pathway	Agent
AKT	mTOR signaling pathway	Everolimus
ERBB2	RTK/MAPK signaling pathway	Lapatanib
EGFR	EGF signaling pathway	Erlotinib
VEGFR	VEGF signaling pathway	Sunitinib
CDK4/6	CDK signaling pathway	Palbociclib
AG‐α3β1	Hedgehog pathway	NA
GLI1/2	Hedgehog pathway	NA
FGFR3	RTK signaling pathway	NA
TP53	p53 signaling pathway	NA
ARID1A	AKT signaling pathway	NA
RB/CDKN1A	Cell‐cycle pathway	NA

NA, Not applicable or not available.

## 4 EPILOGUE AND FUTURE PERSPECTIVES

The molecular typing and curative effect prediction model based on genomics and big data analysis will certainly bring more breakthroughs in precision medicine of bladder cancer. The molecular mechanism of carcinogenesis and progression of bladder cancer should be further investigated to develop robust diagnostic biomarkers and provide experimental basis for bladder cancer targeted therapy. Researchers might make efforts in converting basic research results into clinical application, developing new diagnosis techniques, non‐invasive diagnostic panel, and targeted therapy agents. Moreover, precision medicine of bladder cancer should consider patients’ living circumstances, clinical manifestation, and individual differences, satisfy the complex clinical needs of patients in a more personalized approach and promote the biomedical research development.

## CONFLICT OF INTEREST

None declared.
